# Optical demonstration of crystallography and reciprocal space using laser diffraction from Au microdisc arrays

**DOI:** 10.1107/S1600576721013492

**Published:** 2022-02-01

**Authors:** Lert Chayanun, Johan Gustafson, Jesper Wallentin

**Affiliations:** a Lund University, Box 118, Lund 22100, Sweden

**Keywords:** teaching, education, diffraction, reciprocal space

## Abstract

Crystallography is an invaluable tool in materials science, solid state physics and protein science. Understanding crystallography requires grasping the powerful but abstract concept of reciprocal space. Here a simple but insightful experiment using a laser pointer and Au microdisc arrays to explore and illustrate Bragg diffraction and reciprocal space is demonstrated. The Au microdisc arrays were manufactured using standard semiconductor fabrication techniques. The flexibility of the array design allows the demonstration of basic concepts such as lattice and atomic form factor, but also more advanced ones such as quasicrystal and shape function.

## Abstract 2

A simple experiment to explore and illustrate Bragg diffraction and reciprocal space using a laser pointer and Au microdisc arrays is demonstrated. The flexibility of the array design allows the demonstration of basic concepts such as lattice and atomic form factor, but also more advanced ones such as quasicrystal and shape function.

## 1. Introduction

Crystal diffraction is a vital tool in many fields of science, in particular solid state physics. Any in-depth description of diffraction, beyond Bragg’s law, requires the introduction of reciprocal space, which is also used in many other areas of physics. The concepts of diffraction and reciprocal space are quite challenging for undergraduate students (Radford, 1975 ▸). Laboratory experiments can be a powerful tool to support student learning, but they are difficult to realize in the case of diffraction. Crystallography of atomic crystals is normally performed with X-rays, electrons or neutrons, which is experimentally challenging in an undergraduate teaching context. X-rays require radiation protection, whereas electron beams require a vacuum. The most readily available source of coherent radiation is laser beams, which can be generated by low-cost battery-driven laser pointers. However, the wavelengths of lasers are generally too large for atomic diffraction.

Here, we demonstrate how laser diffraction can be used for student experiments in diffraction and reciprocal space. To carry out an experiment with Bragg diffraction using visible light, the crystal lattice spacing should be in the micrometre range. Optical experiments of diffraction for educational purposes have previously been reported using gratings (Burch *et al.*, 1985 ▸; Logiurato *et al.*, 2020 ▸), overhead projector films (Ferralis *et al.*, 2004 ▸), microdisplays (Lehmann *et al.*, 2019 ▸) and nanowire arrays (Hannibal Madsen *et al.*, 2013 ▸). We used artificial arrays of Au microdiscs on silicon wafers, created with electron beam lithography (EBL). This is a standard method in semiconductor fabrication, and its great flexibility makes it easy to fabricate samples that illustrate various concepts. The required feature sizes are fully compatible with lower-resolution methods such as UV lithography. The experiment presented here is part of an undergraduate course in X-ray physics, based on the textbook by Als-Nielsen & McMorrow (2011 ▸). The laser diffraction experiment is complemented by an X-ray diffraction experiment using metal powders.

## 2. Method

**Figure 1:**
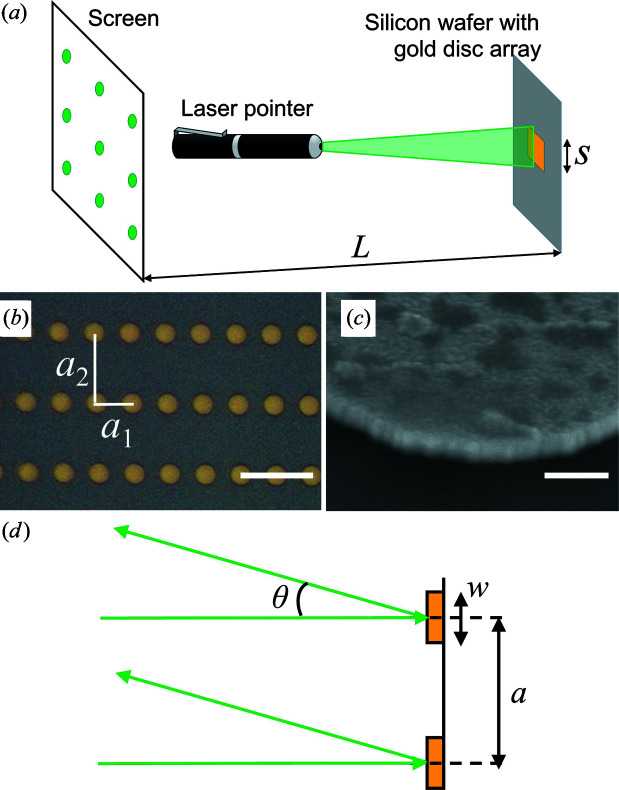
(*a*) Experimental setup to demonstrate the reciprocal space with laser diffraction from an Au microdisc array. (*b*) Microscopy image of an Au microdisc array (scale bar 10 µm). (*c*) Higher-magnification SEM image, tilt 30°, showing the edge of a single microdisc (scale bar 300 nm). (*d*) Schematic of the diffraction geometry.

We used EBL and metal evaporation to create various Au film patterns on a silicon wafer [Fig. 1 ▸(*b*)]. The fabrication process started with spin-coating of the photoresist poly(methyl methacrylate) on the Si wafer. The sample was baked at 453 K for 90 s before EBL exposure. The resist was then developed in a mixture of MIBK:IPA (1:3) for 75 s. After that, layers of Ti and Au were deposited by metal evaporation, with thicknesses of 10 and 190 nm, respectively. Finally, the wafer was soaked in acetone for the lift-off process for 15 min.

A scanning electron microscopy (SEM) image of a typical sample is shown in Fig. 1 ▸(*b*). This particular sample has circular microdiscs with a diameter of *w* = 3 µm and lattice spacings of *a*_1_ = 5 µm and *a*_2_ = 10 µm in the horizontal and vertical directions, respectively. Fig. 1 ▸(*c*) is a high-magnification SEM image of the edge of a microdisc at a 30° tilt, showing a thickness of about 200 nm. Manufacturing structures of this size is straightforward with EBL, but also well within the reach of lower-resolution patterning techniques. The overall size of the pattern is about 0.5–1 mm, in order to fit within the laser spot. Initially, we fabricated the arrays on transparent Si_3_N_4_ membranes, which made it possible to perform the experiment in transmission (Laue) geometry. However, we found that the diffracted intensity was much stronger in reflection geometry.

The samples have features on three different length scales, ranging from the microdisc size, *w*, via the lattice spacings, *a*, to the overall size of the array, *s*. In the diffraction measurements the largest features are observed as the smallest diffraction features, and vice versa, which is an example of the general rule that small features in real space become large in reciprocal space. For the experiment, the students are given a set of labelled samples with unknown patterns, as well as a laser pointer with a holder, a paper screen and a tape measure. Their task is to use laser diffraction to determine the pattern on the samples. As an extra task, the students can use the same experimental approach to quantify the pixel pitch in their mobile phone screens.

The laser pointer was put at an almost normal angle (90°) to the microdisc array, as illustrated in Fig. 1 ▸. We observed that better results were achieved with the laser positioned some distance away from the sample, to have a sufficiently large spot on the sample. The incident beam was then diffracted and projected onto a screen located behind the laser pointer at a distance *L*, typically 1–2 m from the microdisc array [Fig. 1 ▸(*a*)]. By aligning the laser pointer slightly off the normal to the sample, the central (0,0) reflection could be observed and used as a reference. Although many of the Bragg peaks are sufficiently intense to be observed in ambient room light, we used a dark room to be able to also see the fainter Bragg peaks. To capture the details of individual Bragg peaks, a computer-controlled CCD camera with the detector size 22.3 × 14.9 mm (5184 × 3456 pixels) was used. However, a mobile phone camera can also be used for this task.

## 3. Theory

The geometry of the 2D diffraction experiment, shown in Fig. 1 ▸(*d*), is different from regular X-ray diffraction, and it is more similar to low-energy electron diffraction. The diffraction patterns can be analyzed at different levels of sophistication. The simplest model is the grating equation: *m*λ = *a*sinθ, which can be derived by the students themselves. Note that the regular Bragg law is derived for a slightly different geometry.

A more complete model describes the sample as a lattice **R**_*n*_ = *n*_1_ **a**_1_ + *n*_2_ **a**_2_, and the reciprocal lattice 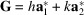 , which is the Fourier transform of the direct lattice. The scattering vector **Q** is the difference between the incident and scattered wavevectors, **Q** = **k** − **k**′. Constructive interference is obtained if the scattering satisfies the Laue condition 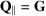 , where 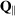 is the component of **Q** which is parallel to the lattice and the surface. This model is sufficient to explain the reciprocal lattice observed in the diffraction patterns.

Additionally, the shapes of the discs can be considered. The scattering amplitude of a crystal is given by 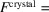 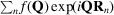 , where *f*(**Q**) is the atomic form factor. In the laser diffraction experiment, the form factor corresponds to the Fourier transform of the shape of the individual discs. Since the lattice spacing *a* is 3–10 times larger than the discs, *w*, *f*(**Q**) will appear at significantly larger length scales in the diffraction patterns. In the artificial arrays, unlike atomic crystals, we can freely control the shape of the discs.

Finally, we can consider the overall shape of the crystal. A finite crystal can be described by its shape function *S*(**r**), which is one in the crystal and zero outside. In a diffraction experiment, each Bragg peak will be convoluted with the Fourier transform of the shape function, *S*(**q**) (Robinson *et al.*, 2001 ▸). Note that this requires the illumination to cover the entire shape. Since the shape of the crystal is much larger than the lattice spacing, in our case about 100 times larger, *S*(**q**) will be much smaller than the reciprocal lattice.

## 4. Results and discussion

**Figure 2:**
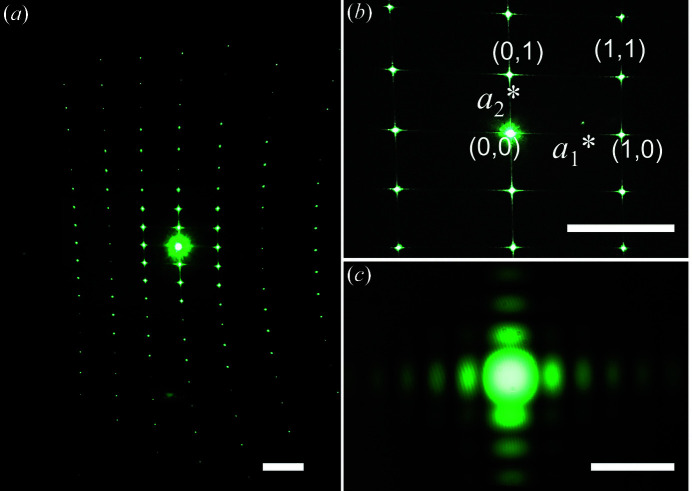
(*a*) Photograph of the long-range diffraction pattern from the microdisc array in Fig. 1 ▸(*b*), acquired in a dark room with a mobile phone camera (scale bar 150 mm). The intense spot in the middle corresponds to the zeroth-order reflection (0,0). (*b*) Higher-resolution photograph of the central area in (*a*) (scale bar 150 mm). (*c*) Diffraction spot (1,1) in (*b*) acquired with a camera (scale bar 5 mm).

We first discuss the results from one sample in some detail, before presenting other samples. Fig. 2 ▸(*a*) shows the diffraction from the Au microdisc array shown in Fig. 1 ▸(*b*), exhibiting features at different length scales. The most characteristic feature is the rectangular diffraction pattern coming from the rectangular array, as shown in Fig. 2 ▸(*b*). The peak distance in the horizontal direction is larger than that in the vertical, since the rectangular lattice is oriented with the smaller distance horizontally. There is a slight curvature, as the flat screen does not follow the Ewald sphere.

The largest observed pattern in Fig. 2 ▸(*a*) is the ring-like pattern originating from the circular Au discs. The form factor *f*(**Q**) is the Fourier transform of the disc shape, meaning that the circular discs will generate an Airy disc pattern. The pattern can be used to estimate the diameter of the discs, *w*, since the angle of the first minimum in an Airy disc pattern is approximately sinθ ≃ 1.22(λ/*w*). A rough estimate, which is incorrect by factor of 1.22, can be made using the grating equation with the diameter of the discs as *a*.

The smallest features are the millimetre-sized fringes observed around each Bragg peak, shown in Fig. 2 ▸(*c*), which are the Fourier transforms of the shape function. The pattern can be observed by eye or with a mobile phone camera, but we used a camera to capture the details. The quadratic shape of the array, not shown here, gives rise to a 2D sinc function. By measuring the fringe positions, the size of the array can be measured. In principle, it should also be possible to use phase retrieval methods to calculate the shape of an arbitrarily shaped array, as is done in Bragg coherent diffractive imaging (Robinson *et al.*, 2001 ▸) and was demonstrated with laser light in transmission geometry (Thibault & Rankenburg, 2007 ▸), but we have not explored this in the course.

**Figure 3:**
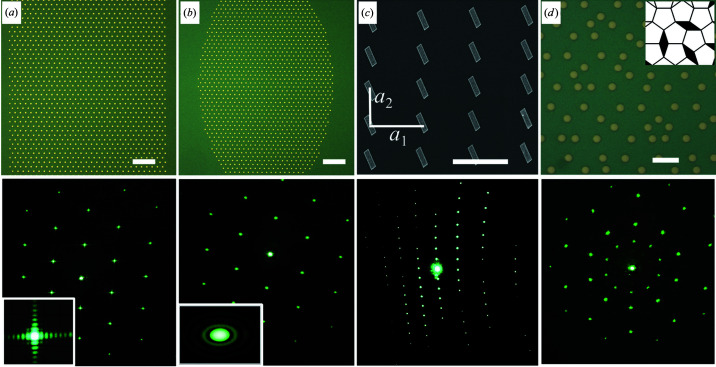
(*a*) Rectangular-shaped array with a hexagonal lattice (scale bar 50 µm). (*b*) Elliptical-shaped array with a hexagonal lattice (scale bar 50 µm). (*c*) SEM showing an array of tilted rectangles (scale bar 10 µm). (*d*) Quasicrystal pattern (scale bar 10 µm). In (*a*)–(*d*) the corresponding diffraction patterns are shown below. The insets in (*a*) and (*b*) show high-resolution photographs of the individual Bragg peaks.

The flexibility of EBL means that the samples can be tailored to illustrate various concepts in diffraction, both artificial and naturally occurring ones. Fig. 3 ▸(*a*) shows a hexagonal lattice in a square-shaped array, and Fig. 3 ▸(*b*) shows an identical lattice in an elliptic array. The corresponding diffraction patterns are shown below, with insets depicting details of single Bragg peaks. In this comparison, the lattices and reciprocal lattices are identical, but the Bragg peaks are different due to the distinct shape functions. The students can use the optical diffraction measurements to identify and estimate the sizes of the arrays.

The shape of the microdiscs can also be changed, as shown in Fig. 3 ▸(*c*). Here, the form factor is tilted rectangles, rather than circular discs, which gives rise to a striped long-range pattern rather than the Airy rings in Fig. 2 ▸(*a*). Note that the diffraction pattern is otherwise the same as in Fig. 2 ▸(*a*), since the lattice was identical. Finally, we created a quasicrystal pattern (Shechtman *et al.*, 1984 ▸, Ferralis *et al.*, 2004 ▸) inspired by a recent report (Wasio *et al.*, 2014 ▸), as shown in Fig. 3 ▸(*d*).

## 5. Conclusion

We have demonstrated the use of a simple experimental setup to explore reciprocal space, which is a crucial concept to interpret the results from diffraction experiments. By measuring the laser diffraction, various aspects of our Au microdisc array can be revealed. We explored various lattice types, lattice spacings, atomic form factors and array shapes. It is also trivial but instructional to demonstrate the effect of the laser wavelength, although we have not shown it here. The same type of experiment could also be used to illustrate more advanced concepts, such as forbidden reflections, strain, twin domains and phase retrieval.
